# Comparative performance of fully-automated and semi-automated artificial intelligence methods for the detection of clinically significant prostate cancer on MRI: a systematic review

**DOI:** 10.1186/s13244-022-01199-3

**Published:** 2022-03-28

**Authors:** Nikita Sushentsev, Nadia Moreira Da Silva, Michael Yeung, Tristan Barrett, Evis Sala, Michael Roberts, Leonardo Rundo

**Affiliations:** 1 Department of Radiology, University of Cambridge School of Clinical Medicine, Addenbrooke’s Hospital and University of Cambridge, Cambridge Biomedical Campus, Box 218, Cambridge, CB2 0QQ UK; 2 Lucida Medical Ltd, Biomedical Innovation Hub, University of Cambridge, Cambridge, UK; 3 Cancer Research UK Cambridge Centre, University of Cambridge, Cambridge, UK; 4 Department of Applied Mathematics and Theoretical Physics, The Cambridge Mathematics of Information in Healthcare Hub, University of Cambridge, Cambridge, UK; 5 Oncology R&D, AstraZeneca, Cambridge, UK; 6 Department of Information and Electrical Engineering and Applied Mathematics (DIEM), University of Salerno, Fisciano, SA Italy

**Keywords:** Prostate cancer, MRI, Artificial intelligence, Deep learning, Machine learning

## Abstract

**Objectives:**

We systematically reviewed the current literature evaluating the ability of fully-automated deep learning (DL) and semi-automated traditional machine learning (TML) MRI-based artificial intelligence (AI) methods to differentiate clinically significant prostate cancer (csPCa) from indolent PCa (iPCa) and benign conditions.

**Methods:**

We performed a computerised bibliographic search of studies indexed in MEDLINE/PubMed, arXiv, medRxiv, and bioRxiv between 1 January 2016 and 31 July 2021. Two reviewers performed the title/abstract and full-text screening. The remaining papers were screened by four reviewers using the Checklist for Artificial Intelligence in Medical Imaging (CLAIM) for DL studies and Radiomics Quality Score (RQS) for TML studies. Papers that fulfilled the pre-defined screening requirements underwent full CLAIM/RQS evaluation alongside the risk of bias assessment using QUADAS-2, both conducted by the same four reviewers. Standard measures of discrimination were extracted for the developed predictive models.

**Results:**

17/28 papers (five DL and twelve TML) passed the quality screening and were subject to a full CLAIM/RQS/QUADAS-2 assessment, which revealed a substantial study heterogeneity that precluded us from performing quantitative analysis as part of this review. The mean RQS of TML papers was 11/36, and a total of five papers had a high risk of bias. AUCs of DL and TML papers with low risk of bias ranged between 0.80–0.89 and 0.75–0.88, respectively.

**Conclusion:**

We observed comparable performance of the two classes of AI methods and identified a number of common methodological limitations and biases that future studies will need to address to ensure the generalisability of the developed models.

**Supplementary Information:**

The online version contains supplementary material available at 10.1186/s13244-022-01199-3.

## Key points

- Fully-automated and semi-automated MRI-based AI algorithms show comparable performance for differentiating csPCa/iPCa.
- DL and TML papers share common methodological limitations discussed in this review.
- Consensus on datasets, segmentation, ground truth assessment, and model evaluation are needed.

## Background

The introduction of pre-biopsy multiparametric magnetic resonance imaging (mpMRI) has considerably improved the quality of prostate cancer (PCa) diagnosis by reducing the number of unnecessary biopsies and increasing the detection of clinically significant disease compared to the conventional PSA-transrectal ultrasound (TRUS) pathway [1–3]. However, the high dependence of the diagnostic performance of mpMRI on reader experience [4, 5] and image quality [6], coupled with the need to balance the time-consuming delineation of biopsy targets against the increasing pressure on radiology departments [7], limits the population-based delivery of high-quality mpMRI-driven PCa diagnosis.

The recent joint position paper by the European Society of Urogenital Radiology (ESUR) and European Association of Urology (EAU) Section of Urological Imaging (ESUI) has highlighted the importance of developing robust and clinically applicable artificial intelligence (AI) methods for overcoming the aforementioned limitations and facilitating the successful deployment of the mpMRI-driven PCa diagnostic pathway [8] to the community. Importantly, the authors suggest the use of AI as a triage tool to detect and delineate areas suspicious for clinically significant PCa (csPCa), where its accurate differentiation from indolent PCa (iPCa) and benign conditions determines the need for subsequent biopsy and defines the diagnostic accuracy of mpMRI. While several recent systematic [9–12] and narrative [13] reviews have described the performance of AI methods for detecting csPCa on MRI, little is known about the comparative performance of fully-automated and semi-automated approaches when applied to this specific clinical task. The rationale for this comparison is based on several inherent differences between the two approaches. Specifically, fully-automated methods rely on learned deep radiomic features and do not require human input following initial training and validation, which underpins their disruptive potential for significantly reducing the radiologists’ clinical workload. Conversely, semi-automated methods, most commonly based on hand-engineered radiomic features, require manual delineation and image pre-processing that may increase the radiologists’ time while not adding significant diagnostic benefit.

Therefore, the primary objective of this systematic review was to analyse the current literature on fully-automated and semi-automated AI methods to differentiate csPCa from iPCa and benign disease on MRI. In addition, we aimed to both identify and offer prospective solutions to common methodological limitations and biases of the existing studies. Addressing these issues going forward will facilitate the development of robust, generalisable, and clinically applicable MRI-derived AI models for PCa diagnosis.

## Materials and methods

To avoid bias, the review protocol was agreed by all authors and registered with PROSPERO (CRD42021270309) before the start of the review process.

### Search strategy

Data collection and reporting were conducted following the Preferred Reporting Items for Systematic Reviews and Meta-Analyses (PRISMA) [14], with a complete PRISMA 2020 checklist presented in Additional file 2: Table S1. We performed a computerised bibliographic search of published and unpublished studies indexed in MEDLINE/PubMed, arXiv, medRxiv, and bioRxiv between 1 January 2016 and 31 July 2021. The full search strategy is summarised in Additional file 1.

### Eligibility criteria

The population of interest included treatment-naïve patients who underwent MRI of the prostate that was subsequently processed using either fully-automated or semi-automated AI methods for lesion detection and subsequent binary classification as (a) csPCa or (b) iPCa or benign disease. The performance of AI methods (index test) was referenced against histopathological assessment of MRI target lesions, with csPCa defined as International Society of Urogenital Pathology (ISUP) grade group ≥ 2 disease and iPCa defined as ISUP grade group 1 disease. The outcome measures included the diagnostic performance of AI approaches for differentiating csPCa from iPCa and benign disease measured as an area under the receiving operator characteristic curve (AUC), sensitivity, specificity, accuracy, positive predictive value (PPV), and negative predictive value (NPV). Only studies written in English and presenting original results were included in this review.

### Systematic review process

We deployed a three-stage process to identify papers suitable for inclusion in this review using Covidence [15] as a Web-based support tool. In the first stage, a team of two reviewers (N.S., L.R.) independently performed the title and abstract screening to ensure relevance, with conflicts resolved by the third reviewer (T.B.). In the second stage, the same two reviewers screened the full text of each paper for eligibility, with conflicts resolved by the same third reviewer. In the third stage, four reviewers (Team 1, N.S., NMDS; Team 2, L.R., M.Y.) evaluated the quality of the documentation of methodologies in the papers to assess the reproducibility of their results. Papers using fully-automated AI methods based on deep learning (DL) methods were assessed using the Checklist for Artificial Intelligence in Medical Imaging (CLAIM) [16], while studies deploying semi-automated AI approaches relying on traditional machine learning (TML) methods were evaluated using the Radiomics Quality Score (RQS) [17] as detailed in Additional file 1.

### Risk of bias assessment

We used the Quality Assessment of Diagnostic Accuracy Studies (QUADAS-2) tool [18] to assess the risk of bias and applicability of studies included in this systematic review. In line with the QUADAS-2 guidance, we developed a review-specific protocol on how to assess each signalling question, which is summarised in Additional file 1. QUADAS-2 assessment was conducted by the same two teams of two reviewers, with each paper reviewed independently by the reviewers prior to conflict resolution by consensus of all four reviewers.

### Data extraction

The data extraction criteria were agreed prior to the review commencement and then independently extracted by the same reviewer teams. The full list of extracted parameters is presented in Additional file 3, with the key diagnostic performance characteristics being AUC, sensitivity, specificity, accuracy, NPV and PPV for the internal holdout or external test sets (when available).

### Data analysis

Given the substantial heterogeneity of patient characteristics, AI algorithms, ground truth assessment methods, and validation strategies used in the diagnostic accuracy studies included in this review, we chose narrative synthesis over meta-analysis of the pooled data to avoid a biased result [19].

## Results

### Study selection

**Fig. 1:**
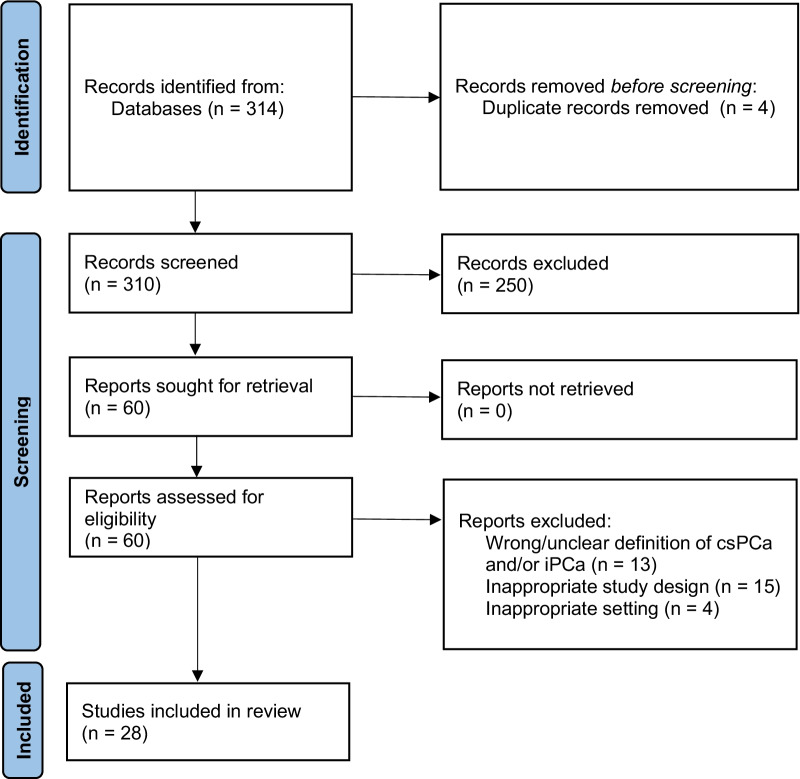
Preferred Reporting Items for Systematic Reviews and Meta-Analyses (PRISMA) 2020 flow diagram for literature search. csPCa, clinically significant prostate cancer; iPCa, indolent prostate cancer

The study selection process is presented in Fig. 1. Our initial search identified 314 papers, of which 4 were highlighted as duplicates by Covidence and removed by us following manual verification. 60/310 papers had titles or abstract deemed relevant to the review question; of those, 28 were retained for quality review after full-text screening. 12/28 papers deployed fully-automated AI methods based on DL methods and were therefore screened using CLAIM, while 16/28 papers used TML methods to develop semi-automated AI approaches and were assessed using RQS. Of these, 5/12 (42%) DL papers [20–24] and 12/16 (43%) TML papers [25–36] passed the quality screening and were subject to full QUADAS-2 assessment, data extraction, and narrative synthesis.

### Quality review

Three out of 12 DL studies (25%) [37–39] that underwent quality screening using CLAIM failed at least three pre-identified mandatory criteria, with 2/12 [40, 41] failing two, and 2/12 [42, 43] failing just one criterion. Four of the seven rejected papers (57%) [37–39, 43] did not describe data processing steps in sufficient detail (Q9), 4/7 [38–40, 42] did not explain the exact method of selecting the final model (Q26), and 3/7 [38, 40, 41] failed to provide enough details on training approach (Q25). Following the subsequent full CLAIM assessment of the remaining five papers, we found that none of them reported the following items: selection of data subsets (Q10), robustness or sensitivity analysis (Q30), validation or testing on external data (Q32), and failure analysis of incorrectly classified cases (Q37). The results of CLAIM quality screening and full assessment are presented in Additional file 1.

One out of 16 TML studies (6%) [44] that underwent quality screening using RQS scored 2/8, 1/16 [45] scored 6/8, and 2/16 [46, 47] scored 7/8, which led to their exclusion from subsequent full RQS assessment. None of the excluded papers had well-documented imaging protocols (Q1) and neither performed multiple segmentations by different radiologists nor conducted robustness analysis of image segmentations to region-of-interest (ROI) morphological perturbations (Q2). The mean RQS of the remaining 12 papers that underwent full assessment was 10.9 ± 2.0 (standard deviation) out of 36 points possible. None of the papers performed phantom studies to detect scanner-dependent features (Q3), reported calibration statistics (Q10), registered a prospective study (Q11), and reported on the cost-effectiveness of the clinical application of the proposed models (Q14). Only one (8%) paper [32] discussed a potential biological correlate for some radiomic features included in the final model (Q7), and only two papers [28, 36] performed external testing of their models (Q12). Furthermore, only six out of 12 (50%) papers [25, 26, 29–32] had image segmentation performed by multiple radiologists or instead assessed the robustness of radiomic features to ROI morphological perturbations (Q2). Eight out of 12 (67%) papers [25–27, 30–32, 34, 35] did not make available any images, code, or feature values used to train the models (Q16), and only 4/12 (33%) papers [30, 31, 34, 36] incorporated non-radiomic features into the multivariable analysis (Q6). The results of RQS screening and full assessment are presented in Additional file 3.

### Risk of bias assessment

**Fig. 2:**
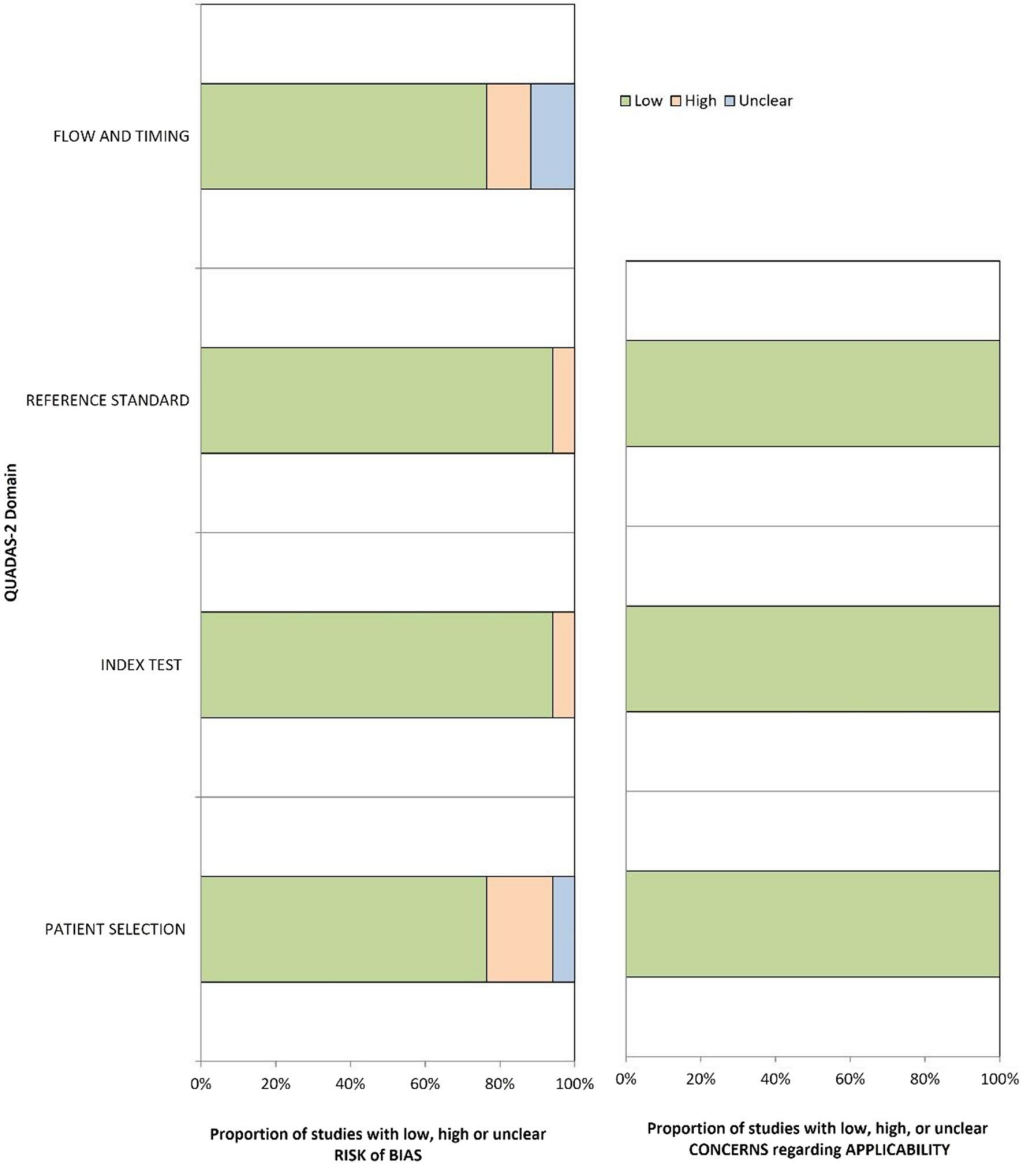
Summary QUADAS-2 risk of bias and applicability concerns assessment

The full results of QUADAS-2 assessment are presented in Additional file 1, with their graphical summary provided in Table 1 and Fig. 2. Overall, 11/17 (65%) [12, 20–23, 25, 26, 29, 31, 34, 36], 1/17 [35], and 5/17 [24, 27, 30, 32, 33] papers had low, unclear, and high risk of bias, respectively. All papers had low applicability concerns. Inappropriate patient selection led to a high risk of bias in 3/5 (60%) studies [27, 30, 33], with two papers containing inappropriate exclusions and one study using a case–control design. One study [30] did not pre-specify a threshold prior to evaluation of the index test performance on the test set. One study [32] used transrectal ultrasound guided (TRUS) biopsy performed six weeks prior to MRI as a reference standard, which introduced a high risk of bias. Two (40%) papers [24, 32] had high risk of bias associated with data flow and timing between the index test (MRI) and reference standard (biopsy), with one paper using both surgical pathology and biopsy results as reference standards, and one paper reporting a six week interval between biopsy and MRI, which was below the recommended threshold of at least six months [48]. The only paper with an unclear risk of bias did not report any information regarding the timing between MRI and biopsy, as well as the specific type of biopsy and whether it was consistent in all patients in the study.

**Table 1:**
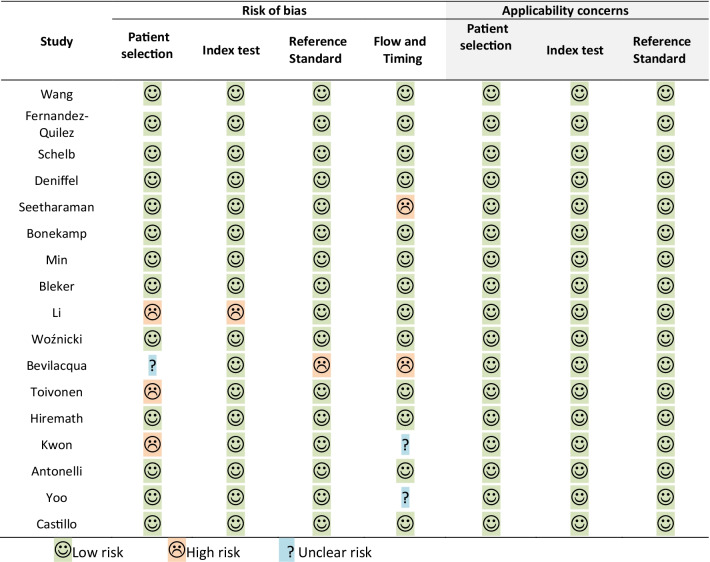
QUADAS-2 risk of bias and applicability concerns

### Study characteristics

Summary demographic characteristics of patients included in the studies that passed the quality screening are presented in Table 2. Two out of five (40%) DL papers [20, 21] used patient data available as part of the open-source PROSTATEx challenge dataset [49], while the remaining three (60%) studies [22–24] used data from single institutions. Importantly, one paper [24] used radical prostatectomy and targeted biopsy interchangeably in one of its patient cohorts. None of the DL studies reported the time between MRI and biopsy, while all studies performed MRI using a single vendor. The number of readers annotating MR images varied between 1 and 4, with reader experience ranging between 0.5 and 20 years.

**Table 2:** Summary demographic characteristics of patients included in the studies selected for narrative synthesis

Study	Year	Country	No. of patients	Age, years	PSA, ng/mL	Patient population	Bx	MRI vs Bx	Time MRI to Bx	No. of centres /vendors	No. of readers	Reader experience, years
*Studies using deep learning-based fully-automated AI methods*												
Wang [20]	2020	Netherlands	346	66 (48–83)	13 (1–56)	Clinically suspected	TB	Pre-Bx	NR	1/1	1	20
Fernandez-Quilez [21]	2021	Netherlands	200	66 (48–83)	13 (1–56)	Clinically suspected	TB	Pre-Bx	NR	1/1	4	NR
Schelb [22]	2019	Germany	312	Training: 64 [58–71] Test: 64 [60–69]	Training: 7.0 [5.0–10.2] Test: 6.9 [5.1–8.9]	Clinically suspected	TB	Pre-Bx	NR	1/1	2	0.5, 10
Deniffel [23]	2020	Canada	499	Training: 63.8 ± 8.1 Test: 64.4 ± 8.4	Training: 7.6 [5.0–10.8]^a^ Test: 7.2 [5.2–11.2]	Clinically suspected	TB	Pre-Bx	NR	1/1	2	15, 3
Seetharaman [24]	2021	USA	424	Training: 63.8 (49–76) Test: 65 (38–82)	Training: 6.8 (3.3–28.6) Test: 7.1 (0.9–63.0)	Clinically suspected	RP or TB	Pre-Bx or Pre-Op	NR	1/1	Unclear	Unclear
*Studies using traditional machine learning-based semi-automated AI methods*												
Bonekamp [25]	2018	Germany	316	64 [58–71]	Training: 6.6 [4.9–9.5] Test: 7.5 [5.4–11.0]	Clinically suspected	TB	Pre-Bx	NR	1/1	2	0.5, 8
Min [26]	2019	China	280	Training,csPCa: 68.8 ± 8.3Training, iPCa: 71.5 ± 8.4 Test, csPCa: 70.3 ± 7.8 Test, iPCa: 71.6 ± 5.7	NR^b^	Clinically suspected	TB	Pre-Bx	NR	1/1	2	NR, 20
Kwon [27]	2018	Netherlands	344	66 (48–83)	13 (1–56)	Clinically suspected	TB	Pre-Bx	NR	1/1	2	> 25
Castillo [28]	2021	Netherlands	107	C1: 64 ± 7 C2: N/A C3: N/A	C1: 12 ± 10 C2: 9 ± 5 C3: 10 ± 8	Clinically suspected	RP	Pre-Op	NR	3/3	1	NR
Bleker [29]	2019	Netherlands	206	66 (48–83)	13 (1–56)	Clinically suspected	TB	Pre-Bx	NR	1/1	Unclear	Unclear
Li [30]	2020	China	381	csPCa:75 [68–81] iPCa: 69 [63–75]	csPCa:49.3 [21.1–83.4 iPCa:9.9 [6.7–15.9]	Clinically suspected	TB	Pre-Bx	NR	1/1	2	3, 9
Woźnicki [31]	2020	Germany	191	Training: 68 [63–74] Test: 69 [63–72]	Training:7.6 [5.7–11.0] Test: 8.2 [6.8–11.9]	Clinically suspected	TB	Pre-Bx	Bx 3 months before MRI	1/2	2	7, 7
Bevilacqua [32]	2021	Italy	76	csPCa: 66 ± 6.8 iPCa: 65 ± 8.8	csPCa: 7.8 ± 7.5 iPCa: 5.3 ± 3.0	Biopsy-proven	TB	Post-Bx	Bx 6 weeks before MRI	1/1	2	7, 25
Toivonen [33]	2019	Finland	62	65 (45–73)	9.3 (1.3–30)	Biopsy-proven	RP	Pre-Bx	NR	1/1	2	NR
Antonelli [34]	2019	UK	164	64 (43–83)	7.4 (2.5–30.3)	Clinically suspected	TB	Pre-Bx	NR	1/1	1	3
Yoo [35]	2019	Canada	427	NR	NR	Clinically suspected	NR	Pre-Bx	NR	1/1	NR	NR
Hiremath [36]	2021	USA, Netherlands	592	C1: 65.5 (59–72) C2: 63 (59–68) C3: 62 (56–66) C4: 65.5 (62–73)	C1: 6.6 (0.25–88.2) C2: 6.7 (5–10) C3: 5.7 (4.54–9.58) C4: 7.7 (4.8–11.3)	Clinically suspected	RP or SB or TB	Pre-Bx	NR	5/3	5	> 15, > 15, > 15, > 10, > 10

Bx, biopsy; C, cohort; MRI, magnetic resonance imaging; NR, not reported; PSA, prostate-specific antigen; RP, radical prostatectomy; SB, systematic biopsy; TB, targeted biopsy

^a^Data missing for 110 cases

^b^PSA values were reported by subcategories (< 4 ng/mL, 4–10 ng/mL, > 10 ng/mL), see the original reference [26] for further details

Ten out of 12 (83%) TML papers [12, 25, 26, 30–36] utilised non-publicly available institutional datasets, with the remaining 2/12 (17%) studies [27, 29] using the PROSTATEx challenge dataset [49]. In eight (67%) papers [25–27, 29–32, 34], the histopathological ground truth was obtained using targeted biopsy, while two studies [28, 33] relied on radical prostatectomy data, one [36] was a multi-institutional study relying on either biopsy (targeted or systematic) or prostatectomy data in different cohorts, and one [35] did not explicitly report the source of ground truth. Only two (17%) papers [31, 32] reported the time between biopsy and MRI; in these studies, biopsy was performed either three months [31] or six weeks [32] prior to MRI. Nine (75%) studies [25–27, 29, 30, 32–35] had one centre and one vendor each, while the remaining three studies [28, 31, 36] were multi-vendor. The number of readers varied between 1 and 5, with reader experience ranging between 0.5 and more than 25 years.

### Predictive modelling characteristics

Summary predictive modelling characteristics of DL papers are presented in Table 3. All studies used different convolutional neural network (CNN) architectures, with 3/5 (60%) studies [20, 23, 24] proposing their own networks and 2 papers using off-the-shelf networks, including VGG16 [21] and U-Net [22]. None of the papers included non-imaging features for the purposes of predictive modelling and conducted external testing of the developed predictive models. All DL papers were designed as a classification task to distinguish csPCa from iPCa and benign lesions. Four (80%) studies [21–24] performed the analysis at the level of the whole prostate, and one study [20] separately analysed peripheral and transition zone lesions. Importantly, none of the DL studies validated their results using external datasets.

**Table 3:** Predictive modelling characteristics of studies using deep learning-based fully-automated AI methods

Study	No. of patients	Training set	Validation set	Test set	Algorithm	MRI input	Image registration	Image segmentation	Outcome	Zone	Analysis	Evaluation strategy
Wang [20]	346	204	fivefold CV	142	CNN (MISN)	ADC, BVAL, DWI_0_, DWI_1_, DWI_2_, *K*^trans^, T2WI-Cor, T2WI-Sag, T2WI-Tra	NR	Open data	csPCa vs iPCa or benign lesions	PZ or TZ	Per lesion	Internal hold-out
Fernandez-Quilez [21]	200	NR^a^	NR^a^	NR^a^	CNN (VGG16)	T2WI, ADC	NR	Open data	csPCa vs iPCa or benign lesions	WP	Per lesion	Internal hold-out
Schelb [22]	312	250	No	62	CNN (U-Net)	T2WI, DWI	SimpleITK, non-rigid Bspline with Mattes mutual information criterion	Automated (U-Net)	csPCa vs iPCa or benign lesions	WP	Per lesion, per patient	Internal hold-out
Deniffel [23]	499	324	75	50^b^	CNN (3D)	T2WI, ADC, DWI	Static, affine	Manual bounding boxes	csPCa vs iPCa or benign lesions	WP	Per patient	Internal hold-out
Seetharaman [24]	424	102	fivefold CV	322	CNN (SPCNet)	T2WI, ADC	Manual	Registration from pathology images	csPCa vs iPCa or benign lesions	WP	Per pixel, per lesion	Internal hold-out

ADC, apparent diffusion coefficient; CNN, convolutional neural networks; csPCa, clinically significant prostate cancer; CV, cross-validation; DWI, diffusion-weighted imaging; iPCa, indolent prostate cancer; MISN, multi-input selection network; MRI, magnetic resonance imaging; NR, not reported; PZ, peripheral zone; T2WI, T_2_-weighted imaging; TZ, transition zone; WP, whole prostate

^a^The study included 200 patients and 299 lesions, of which 70% were used to train train, 20% to test, 10% to fine-tune the models

^b^Describes the calibration cohort

Similar predictive modelling characteristics of TML papers are summarised in Table 4. The three most commonly used ML models included random forests (50% papers), logistic regression (42% papers), and support vector machines (25% papers), with 7/12 studies testing several different models. Imaging features were extracted from apparent diffusion coefficient maps, T_2_-weighted images, and diffusion-weighted images with different b-values in 12/12 (100%) [25–36], 9/12 [25–31, 33, 36], and 7/12 [25–29, 32, 35] papers, respectively. In contrast to the DL papers, only 7/12 (58%) TML studies [25, 27, 29–31, 35, 36] differentiated csPCa from iPCa and benign lesions, whereas the remaining five studies (42%) [26, 28, 32–34] did not include benign disease, thereby focusing only on distinguishing csPCa from iPCa. Eight (67%) papers [26, 28, 30–33, 35, 36] performed the analysis at the level of the whole prostate, two [27, 34] reported the results for peripheral and transition zone lesions separately, one [25] developed models for the whole prostate as well as peripheral and transition zone lesions, and one [29] included peripheral zone tumours only. Seven (58%) studies [25–27, 29–32] validated their results using internal hold-out, three papers [33–35] used cross-validation, and the remaining two studies [28, 36] used either a mixed hold-out cohort or a fully external hold-out dataset.

**Table 4:** Predictive modelling characteristics of studies using traditional machine learning-based semi-automated AI methods

Study	No. of patients	Training set	Validation set	Test set	Algorithm	MRI input	IR	IS	Discriminative features	No. of features used for training	Outcome	Zone	Analysis	Evaluation strategy
Bonekamp [25]	316	183	NR	133	RF	T2WI, ADC, b = 1500	No	Manual	First-order, volume, shape, texture	NR	csPCa vs iPCa or benign lesions	WP or PZ or TZ	Per lesion and per patient	Internal hold-out
Min [26]	280	187	NR	93	LR	T2WI, ADC, b = 1500	No	Manual	Intensity, shape, texture, wavelet	9	csPCa vs iPCa	WP	Per lesion	Internal hold-out
Kwon [27]	344	204	tenfold CV	140	CART, RF, LASSO	T2WI, DWI, ADC, DCE	Rigid	No	Intensity	54	csPCa vs iPCa or benign lesions	PZ or TZ	Per lesion	Internal hold-out
Castillo [28]	107	80%	20% of training (100 random repeats)	20%	LR, SVM, RF, NB, LQDA	T2WI, DWI, ADC	HP^a^	Manual	Shape, local binary patterns, GLCM	NR	csPCa vs iPCa	WP	Per lesion, Per patient	Mixed hold-out
Bleker [29]	206	130	NR	76	RF, XGBoost	T2WI, b = 50, b = 400, b = 800, b = 1400, ADC, *K*^trans^	No	Manual	Intensity, texture	NR	csPCa vs iPCa or benign lesions	PZ	Per lesion	Internal hold-out
Li [30]	381	229	NR	152	LR	T2WI, ADC	No	Manual	Intensity, age, PSA, PSAd	15	csPCa vs iPCa or benign lesions	WP	Per lesion	Internal hold-out
Woźnicki [31]	191	151	fivefold CV	40	LR, SVM, RF, XGBoost, CNN	T2WI, ADC	No	Manual	Intensity, shape, PI-RADS, PSAd, DRE	15	csPCa vs iPCa or benign lesions	WP	Per patient	Internal hold-out
Bevilacqua [32]	76	48	threefold CV	28	SVM	ADC, b = 2000	No	Manual	Intensity	10	csPCa vs iPCa	WP	Per lesion	Internal hold-out
Toivonen [33]	62	62	LPOCV	N/A	LR	T2WI, ADC, *K*^trans^, T2 map	No	Manual	Intensity, Sobel, texture	NR	csPCa vs iPCa	WP	Per lesion	LPOCV
Antonelli [34]	164	134	NR	30	PZ: LinR TZ: NB	ADC, DCE	Rigid	Manual	Texture, PSAd	NR	csPCa vs iPCa	PZ or TZ	Per lesion	fivefold CV
Yoo [35]	427	271	48	108	CNN, RF	ADC, DWI	No	No	First-order statistics of deep features	90	csPCa vs iPCa or benign lesions	WP	Per slice, Per patient	tenfold CV
Hiremath [36]	592	368	threefold CV	224	AlexNet or DenseNet and Nomogram	T2WI, ADC	Rigid, affine	Manual	Deep learning imaging predictor, PI-RADS, PSA, gland volume, tumour volume	NR	csPCa vs iPCa or benign lesions	WP	Per patient	External hold-out

ADC, apparent diffusion coefficient; CART, classification and regression trees; CNN, convolutional neural networks; GLCM, grey level co-occurrence matrix; HP, histopathology; IR, image registration; IS, image segmentation; LASSO, least absolute shrinkage and selection operator; LinR, linear regression; LQDA, linear and quadratic discriminant analysis; LR, logistic regression; NB, naïve Bayes; PI-RADS, prostate imaging-reporting and data system; PSA, prostate-specific antigen; PSAd, prostate-specific antigen density; RF, random forests; SVM, support-vector machines

^a^Histopathology images registered with T_2_-weighted images using specialised software

### Comparative performance of fully-automated and semi-automated AI methods

Three out of 5 (60%) DL studies [21–23] had clearly defined thresholds at which performance characteristics of the developed models were calculated; these are presented in Table 5. For studies combining peripheral and transition zone lesions for classification [21, 23, 24], the AUCs of the best-performing models reported in the test sets for differentiating csPCa from iPCa and benign disease ranged between 0.80 and 0.89. Importantly, the AUC range changed to 0.85–0.89 when a study by Seetharaman et al. [24] was excluded from the calculation due to its high risk of bias reported on QUADAS-2 assessment (Table 1). In a study by Wang et al. [20], AUCs for peripheral zone and transition zone lesions were 0.89 [0.86–0.93] and 0.97 [0.95–0.98], respectively, and a study by Schelb et al. [22] did not report AUC values. Four (80%) studies [21–24] did not report accuracy of the developed models, while Wang et al. [20] reported accuracy of 0.91 [0.86–0.95] and 0.89 [0.87–0.91] in the peripheral and transition zone lesions, respectively. All studies reported sensitivity and specificity of the proposed models, while only 2/5 (40%) [22, 23] studies presented NPV and PPV, with NPV being higher in both cases (Table 5).

Six out of 12 (50%) TML studies [25, 30–32, 34, 36] defined specific thresholds for diagnostic performance, with the resulting characteristics summarised in Table 5. The AUCs of the best-performing models for studies combining peripheral and transition zone lesions ranged between 0.75 and 0.98. The AUC range changed to 0.75–0.88 when five papers [27, 30, 32, 33, 35] with high or unclear risk of bias on QUADAS-2 (Table 1) were removed from the calculation. A study by Li et al. [30] (high risk of bias, see Table 1) was one of two papers reporting accuracy of the proposed model (0.90), in addition to a study by Hiremath et al. [36] where it reached 0.78; both studies applied their models to peripheral and transition zone lesions combined. 3/12 (25%) [27, 33, 35] papers did not report sensitivity and specificity of their models, and only one study by Li et al. [30] presented NPV and PPV of their model.

**Table 5:** Diagnostic performance of fully-automated and semi-automated AI methods for differentiating between csPCa and iPCa or benign disease

Study	Threshold	AUC [95% CI]	Accuracy	Sensitivity	Specificity	NPV	PPV
*Studies using deep learning-based fully-automated AI algorithms*							
Wang [20]	NR	PZ: 0.89 [0.86–0.93] TZ: 0.97 [0.95–0.98]	PZ: 0.91 [0.86–0.95] TZ: 0.89 [0.87–0.91]	PZ: 0.60 [0.52–0.69] TZ: 1.0 [1.0–1.0]	PZ: 0.98 [0.95–1.0] TZ: 0.88 [0.82–0.93]	NR	NR
Fernandez-Quilez [21]	0.5	0.89	NR	0.85	0.94	NR	NR
Schelb [22]	Several for different PI-RADS cut-offs	NR	NR	PI-RADS ≥ 3: 0.96 PI-RADS ≥ 4: 0.92	PI-RADS ≥ 3: 0.31 PI-RADS ≥ 4: 0.47	PI-RADS ≥ 3: 0.84 PI-RADS ≥ 4: 0.83	PI-RADS ≥ 3: 0.53 PI-RADS ≥ 4: 0.67
Deniffel [23]	Risk of csPCa ≥ 0.2	0.85 [0.76–0.97]	NR	1.0 [1.0–1.0]	0.52 [0.32–0.68]	1.0 [1.0–1.0]	0.56 [0.48–0.66]
Seetharaman^a^ [24]	NR	0.80 (per lesion)	NR	0.70 (per lesion)	0.77 (per lesion)	NR	NR
*Studies using traditional machine learning-based semi-automated AI algorithms*							
Bonekamp [25]	0.79	WP: 0.88 PZ: 0.84 TZ: 0.89 (per lesion)	NR	WP: 0.97 (per lesion)	WP: 0.58 (per lesion)	NR	NR
Min [26]	NR	0.82 [0.67–0.98]	NR	0.84	0.73	NR	NR
Castillo [28]	NR	0.75	NR	0.88	0.63	NR	NR
Bleker [29]	NR	0.87 [0.75–0.98]	NR	0.86	0.73	NR	NR
Woźnicki [31]	0.45	0.84 [0.6–1.0]	NR	0.91 [0.81–0.98]	0.57 [0.38–0.74]	NR	NR
Antonelli [34]	Reader SP (training)	PZ: 0.83 TZ: 0.75	NR	PZ: 90 TZ: 92	PZ: 65 TZ: 56	NR	NR
Hiremath [36]	Maximising accuracy (0.361)	0.81 [0.76–0.85]	0.78	0.83	0.59	NR	NR
Kwon^a^ [27]	NR	WP: 0.82	NR	NR	NR	NR	NR
Li^a^ [30]	− 0.42	0.98 [0.97–1.00]	0.90	0.95	0.87	0.97	0.82
Bevilacqua^a^ [32]	0.58	0.84 [0.63–0.90]	NR	0.9	0.75	NR	NR
Toivonen^a^ [33]	NR	0.88 [0.92–0.95]	NR	NR	NR	NR	NR
Yoo^a^ [35]	NR	0.84 [0.76–0.91]	NR	NR	NR	NR	NR

AUC, area under the receiver operating characteristic curve; NPV, negative predictive value; NR, not reported; PI-RADS, prostate imaging-reporting and data system; PPV, positive predictive value; PZ, peripheral zone; SP, specificity; TZ, transition zone; WP, whole prostate

^a^These papers had either high or unclear risk of bias on QUADAS-2 assessment (see Table 1; Fig. 2)

## Discussion

This systematic review highlights the intensity of research efforts in developing both fully-automated and semi-automated MRI-derived AI methods for differentiating csPCa from iPCa and benign disease. While formal meta-analysis and direct comparison of the two approaches were not possible due to a substantial heterogeneity of studies included in this review, the narrative synthesis revealed their comparable performance that was marginally higher for fully-automated methods. If common methodological limitations outlined in this review are addressed, future studies will have the potential to make AI-driven expert-level prostate MRI assessment widely accessible and reproducible among multiple centres and readers with different experiences.

In keeping with this report, previous systematic and narrative reviews investigating the diagnostic performance of DL- and TML-based AI methods for PCa diagnosis [9, 11–13] have also highlighted substantial heterogeneity and poor reproducibility of the developed predictive models. While a meta-analysis by Cuocolo et al*.* [10] showed higher AUC of TML-based models compared to DL-based models, the authors drew the data from all studies included in the qualitative synthesis. Some of these studies had a high risk of bias and showed important differences among their patient populations, ground truth assessment methods, zonal distribution of predictive models, and other potential confounders. In our review, the addition of full CLAIM and RQS quality evaluation to QUADAS-2 assessment highlighted high methodological heterogeneity of both DL- and TML-based studies, which limited the reliability of their quantitative synthesis. The outcomes of qualitative synthesis, however, suggest that DL-based fully-automated AI methods may prove more clinically useful in the long run given their comparable performance to TML-based semi-automated methods. A crucial practical advantage of fully-automated approaches is its potential time-saving effect that is important in the context of an ever-increasing workload in radiology departments. That said, almost all DL papers included in this review still require even minimal manual interaction from the readers, including lesion identification as patches [20] or bounding boxes [22–24], thereby still introducing a known element of interobserver variability. However, a head-to-head comparison of DL- and TML-based AI methods in the same patient cohort presents a highly important area of unmet research need. If addressed, this has the capacity to directly answer the clinical question behind this review.

In this review, a combination of full CLAIM, RQS, and QUADAS-2 assessment revealed several common methodological limitations, some of which are applicable to both DL and TML studies. These common limitations fall into four distinct domains: (1) datasets used for model development, (2) methods used to ensure quality and reproducibility of image segmentation, (3) ground truth assessment methods, (4) strategies used for model evaluation. The following paragraphs summarise the key limitations within each of the four domains, with detailed recommendations for their prospective mitigation provided in Additional file 1.

First, the overwhelming majority of papers included in this review either utilised non-publicly available single-centre datasets or used the same open-source single-centre PROSTATEx challenge dataset [49]. The use of single-centre datasets, both public and private, without external testing presents a critical limitation to the clinical applicability of the developed models. Conversely, the use of a single public dataset without additional data encourages community-wide overfitting that limits the utility of the dataset itself.

Second, nearly half of the studies did not process images segmented by multiple radiologists, thus limiting the generalisability of the developed predictive models due to known interobserver variability even among experts [50–52]. The same applies to the original PROSTATEx dataset [49] that includes lesion coordinates based on the outlines provided by a single reader. While one DL study included in our review [20] used the original single-reader segmentations, another study [21] overcame this limitation by utilising segmentations validated by several readers in a dedicated study by Cuocolo et al. [53]. Even if trained on the same dataset and using the same AI methods, models developed using different segmentations will inevitably differ in their performance, which brings additional layer of heterogeneity to the field.

Third, only 80% of DL and 67% of TML papers used MRI-targeted biopsy specimens as a source of ground truth. The remaining studies either relied on radical prostatectomy data or included mixed patient cohorts where the ground truth was obtained using different methods. While radical prostatectomy specimens offer definitive assessment of lesion morphology, the resulting predictive models will have very limited clinical applicability due to overrepresentation of patients with intermediate-risk disease. If predictive models are trained to differentiate between iPCa and csPCa and therefore help clinicians decide on the need for subsequent biopsy, then MRI-targeted biopsy using cognitive, US/MRI fused, or in-bore approaches present an appropriate standard for ground truth assessment.

Fourth, none of the DL papers and only two TML papers used external testing to assess the generalisability of the developed predictive models [54]. Given the intrinsically low reproducibility and repeatability of MRI-derived radiomic features [55, 56], the lack of robust external testing and prior assessment of feature robustness to scanning parameters present major obstacles to the clinical use of any MRI-based AI algorithms. However, even if external testing becomes the norm, it is also important to avoid common mistakes in reporting standard measures of discrimination that help evaluate model performance. These often include the lack of clearly identified operating points at which they were calculated and confidence intervals that reflect the uncertainty in the estimate. Ideally, the operating points should reflect the expected performance of expert radiologists, with the pooled NPV of 97.1% (95% CI 94.9–98.7%) [2] being the key clinical benchmark that has established mpMRI as a diagnostic test that can effectively rule out csPCa. Importantly, a thorough failure analysis of incorrectly classified cases is key to understanding and communicating diagnostic pitfalls of the developed models, which is paramount to their safe and evidence-based clinical use. Finally, despite pointing out the above pitfalls, we acknowledge the overall high quality of publications in the field of applying AI methods to mpMRI-driven PCa diagnosis. Improving their methodological quality, the next steps will require a consolidated international and multi-institutional effort, the success of which will primarily depend on the quality of data used for training and validating AI algorithms.

This review has several limitations. The introduction of stringent CLAIM and RQS methodological screening led to the exclusion of several high-quality papers published in high-impact journals, such as *Journal of Magnetic Resonance Imaging*, *European* Radiology, and *Cancers*. This approach, which we previously adopted for another review [57], allowed us to only include studies that are reproducible. It is, however, important to acknowledge that the CLAIM requirements are harder to fulfil compared to the RQS ones. We also acknowledge that some relevant studies may not have been included, particularly those published between our search and publication of this review. Due to the considerable heterogeneity of studies, we did not pool the data for a formal comparison of the diagnostic accuracy of fully-automated and semi-automated AI methods. This was, however, compensated by an extensive narrative synthesis that identified common pitfalls and inconsistencies of the included studies that formed the basis of their heterogeneity.

## Conclusions

In conclusion, we observed comparable performance of fully-automated and semi-automated MRI-derived AI methods for differentiating csPCa from iPCa and benign disease. In-depth CLAIM and RQS methodological quality assessment of the studies included in this review revealed several important pitfalls that limit clinical applicability and generalisability of the vast majority of the proposed predictive models. These include, but are not limited to, the use of single-centre datasets without external test cohorts, lack of multi-reader image segmentation, use of inappropriate ground truth assessment methods, and insufficient reporting of model evaluation metrics that can inform their interpretability and clinical applicability. Future studies that address these limitations will help to unlock the disruptive potential of AI and harness the benefits of expert-quality mpMRI-driven PCa diagnosis for the wider community.

### Supplementary Information

**Additional file 1.** Master document summarising the review guidance and results of CLAIM, RQS, and QUADAS-2 assessment.

**Additional file 2.** PRISMA-2020 checklist.

**Additional file 3.** Supplementary Methods.

## Data Availability

All data generated or analysed using this study are included in this published article and its supplementary information files.

## Abbreviations

ADC: Apparent diffusion coefficient
AI: Artificial intelligence
AUC: Area under the receiver operating characteristic curve
CART: Classification and regression trees
CLAIM: Checklist for Artificial Intelligence in Medical Imaging
CNN: Convolutional neural network
csPCa: Clinically significant prostate cancer
DL: Deep learning
GLCM: Grey level co-occurrence matrix
HP: Histopathology
iPCa: Indolent prostate cancer
LASSO: Least absolute shrinkage and selection operator
LinR: Linear regression
LQDA: Linear and quadratic discriminant analysis
LR: Logistic regression
mpMRI: Multiparametric MRI
NB: Naïve Bayes
NPV: Negative predictive value
NR: Not reported
PCa: Prostate cancer
PI-RADS: Prostate imaging–reporting and data system
PPV: Positive predictive value
PSA: Prostate-specific antigen
PSAd: Prostate-specific antigen density
PZ: Peripheral zone
QUADAS-2: Quality Assessment of Diagnostic Accuracy Studies
RF: Random forests
RQS: Radiomics quality score
SVM: Support-vector machine
TML: Traditional machine learning
TZ: Transition zone
WP: Whole prostate
