# Supplementary material for: Comparative performance of fully-automated and semi-automated artificial intelligence methods for the detection of clinically significant prostate cancer on MRI: a systematic review
Source: Insights Imaging. 2022 Mar 28;13:59. doi: 10.1186/s13244-022-01199-3 (PMC8960511; doi:10.1186/s13244-022-01199-3)
Supplement: Supplementary file 3 — Additional file 3. Supplementary Methods. [file 13244_2022_1199_MOESM3_ESM.docx]

**Comparative Performance of Fully-Automated and Semi-Automated Artificial Intelligence Methods for the Detection of Clinically Significant Prostate Cancer on MRI: a Systematic Review**

**Supplementary Information**

**Supplementary Methods**

**Literature search criteria**

PMquery="(prostatic[ti] OR prostate[ti] OR prostatic[ab] OR prostate[ab]) AND (neoplasms[ti] OR cancer[ti] OR neoplasms[ab] OR cancer[ab]) AND (\"magnetic resonance\"[ti] OR mri[ti] OR \"magnetic resonance\"[ti] OR mri[ti] OR mr[ti] OR \"magnetic resonance\"[ab] OR mri[ab] OR \"magnetic resonance\"[ab] OR mri[ab] OR mr[ab]) AND (\"artificial intelligence\"[ti] OR \"deep learning\"[ti] OR \"machine learning\"[ti] OR ai[ti] OR \"artificial intelligence\"[ab] OR \"deep learning\"[ab] OR \"machine learning\"[ab] OR ai[ab]))"

**Systematic review process**

To ensure that only papers with the highest-quality documentation of methodology were included in the review, we excluded papers that failed to fulfil several CLAIM or RQS requirements during the initial quality screening [18]. Specifically, we deemed nine CLAIM checkpoint items mandatory for allowing the reproduction of the study methodology and results. Papers assessed using RQS had to reach a threshold score of 8/36 that was comprised of five mandatory items. The list of mandatory items for both checklists, alongside the rationale for selecting them, is presented in **Supplementary Information (File 1)**. For RQS, the minimum score of 8 was possible if a paper got at least 1, 1, 3, 1, and 2 points (sum = 8) for the mandatory RQS criteria 1, 2, 5, 9, and 12, respectively (see **Supplementary Information (File 1)**, with the choice of the criteria dictated by their critical role for basic reproducibility of the key study results. Only those papers that passed the initial quality screening were eligible for subsequent full CLAIM and RQS scoring, risk of bias assessment, and data extraction. Throughout this stage of the review, the reviewers independently scored each paper against the appropriate checklist, with conflicts subsequently resolved by consensus of all four reviewers. To ensure complementary expertise throughout the review process, reviewers with a clinical background were paired with non-clinicians with a machine learning background.

**Data extraction parameters**

The overall sample size and the number of patients in the training, validation, and test sets, patient age and PSA, patient population characteristics (clinically suspected or biopsy-proven PCa), temporal relationship between MRI and biopsy (including the time between the two tests), magnet strength, number of centres and vendors, type of MRI protocol (multiparametric or biparametric), type of biopsy, reader experience, AI algorithm used for predictive modelling, MRI input sequences, type of image registration, type of predictive analysis (per lesion or per patient), type of discriminative features, number of radiomic features used for training, evaluation strategy, outcome, zonal distributions of lesions (whole prostate or peripheral and transition zones analysed individually), threshold for AUC calculation, as well as AUC, sensitivity, specificity, accuracy, NPV and PPV for the internal holdout or external test sets (when available).

**Supplementary Discussion**

*Recommendations for data collection and processing*

Of the papers included in this review, 60% of DL and 83% of TML studies utilised non-publicly available institutional datasets, and 86% of which were derived from a single institution; the remaining studies all used the same open-source single-centre PROSTATEx challenge dataset [1]. The use of single-centre datasets, both public and private, without external testing presents a critical limitation to the clinical applicability of the developed models. Conversely, the use of a single public dataset without additional data encourages community-wide overfitting that limits the utility of the dataset itself. While recommendations for tackling the challenges associated with external testing are discussed later, the issue of overfitting on the PROSTATEx dataset may be addressed by including studies from other centres and vendors. Such expansion may be limited to one patient population (e.g. biopsy-naïve), which can allow for tailoring the predictive modelling to specific sub-populations within it (e.g. younger and older patients with lower and higher tolerance to false-positive results, respectively). Alternatively, one may choose to expand the dataset by including currently under-represented patient groups (e.g. prior negative or equivocal MRI scans) to make the resulting models more applicable to the wider population presenting with suspected PCa. Regardless of the selected approach, the resulting datasets and studies utilising them should explicitly report the disease prevalence, which is critical to understanding the clinical applicability of the developed models [2]. Importantly, ideal datasets should include all mpMRI sequences performed in all planes, which will enable the development and subsequent comparison of bpMRI and mpMRI predictive models, mirroring the ongoing clinical studies comparing the clinical utility of the two protocols [3–5]. Other important features of high-quality public datasets related to image segmentation and ground truth assessment are summarised below.

*Recommendations for image segmentation*

A sizeable number of studies included in this review (40% of DL papers and 50% of TML papers) neither had MR images segmented by multiple radiologists nor compensated for it by assessing the robustness of MRI-derived radiomic features to simulated ROI morphological perturbations. Importantly, the original PROSTATEx dataset [1] includes lesion coordinates based on the outlines provided by a single reader, which limits the generalisability of the developed predictive models due to known interobserver variability even among experts [6–8]. While one DL study included in our review [9] used the original single-reader segmentations, another study [10] overcame this limitation by utilising segmentations validated by several readers in a dedicated study by Cuocolo *et al*. [11]. Even if trained on the same dataset and using the same AI methods, models developed using different segmentations will inevitably differ in their performance, which brings additional layer of heterogeneity to the field. Going forward, to ensure reliable model performance, training should be based on segmentations provided by multiple expert readers [12, 13], with the performance benchmarked against a wider group of specialised, general, and trainee radiologists [2]. While the developed models are unlikely to significantly outperform expert readers, they can add value to routine clinical practice if proven to have superior performance to less experienced readers who they are being designed for.

*Recommendations for ground truth assessment*

In this review, 80% of DL and 67% of TML papers used MRI-targeted biopsy specimens as a source of ground truth. The remaining studies either relied on radical prostatectomy data or included mixed patient cohorts where the ground truth was obtained using different methods. While radical prostatectomy specimens offer definitive assessment of lesion morphology, the resulting predictive models will have very limited clinical applicability due to overrepresentation of patients with intermediate-risk disease. If predictive models are trained to differentiate between iPCa and csPCa and therefore help clinicians decide on the need for subsequent biopsy, then biopsy results present an appropriate standard for ground truth assessment. In terms of the optimal type of biopsy used for model development, MRI-targeted biopsy using cognitive, US/MRI fused, or in-bore approaches should be prioritised since it offers superior rate of csPCa detection and reduces detection of iPCa compared to systematic TRUS biopsy [14]. Since it may be difficult for individual centres to collect large enough datasets with MRI-targeted biopsy as a reference standard, this is an area where establishing high-quality multi-centre open-source datasets seems to offer the greatest potential.

*Recommendations for evaluation*

In this review, none of the DL papers and only two TML papers used external testing to assess generalisability of the developed predictive models [15]. Given the intrinsically low reproducibility and repeatability of MRI-derived radiomic features [16, 17], the lack of robust external testing and prior assessment of feature robustness to scanning parameters present major obstacles to the clinical use of any MRI-derived AI algorithms. While the conduct of multi-centre studies is often obstructed by ethical and legal constraints, the recent development of federated learning [18] overcomes the barriers to data sharing by training AI models on disparate data sources and merging the results on a federated server that never sees the original data. However, even if external testing becomes the norm, it is important to avoid common mistakes in reporting standard measures of discrimination that help evaluate model performance. These often include the lack of clearly identified operating points at which they were calculated and confidence intervals that reflect the uncertainty in the estimate. Ideally, the operating points should mirror the expected performance of expert radiologists, with the pooled NPV of 97.1% (95% CI 94.9-98.7%) [19] being the key clinical benchmark that has established mpMRI as a diagnostic test that can effectively rule out csPCa. In addition, reporting calibration statistics and decision curve analysis offers a more holistic assessment of the model performance and allows for evaluating predictive error and clinical “net benefit” of the model under consideration [20]. Furthermore, providing saliency maps overlaid onto clinical images and highlighting the key features and areas that most influenced the model decision-making is another important step towards improving clinical interpretability of MRI-derived AI methods. Moreover, a thorough failure analysis of incorrectly classified cases is key to understanding and communicating diagnostic pitfalls of the developed models, which is paramount to their safe and evidence-based use in routine clinical practice. While performing and reporting these evaluation strategies undoubtedly adds several layers of complexity, it is essential to ensure that limited financial and human resources are invested in developing robust models that have high probability of making a real clinical impact.

In addition to the aforementioned recommendations, we also suggest that authors assess their papers against CLAIM [16], RQS [17], and QUADAS-2 [19] as appropriate. Failure to report essential methodological elements makes reproducibility of the key results virtually impossible, while also undermining the transparency and trustworthiness of the presented data.

**Supplementary References:**

1. Litjens G, Debats O, Barentsz J, et al (2014) Computer-aided detection of prostate cancer in MRI. IEEE Trans Med Imaging. https://doi.org/10.1109/TMI.2014.2303821

2. Penzkofer T, Padhani AR, Turkbey B, et al (2021) ESUR/ESUI position paper: developing artificial intelligence for precision diagnosis of prostate cancer using magnetic resonance imaging. Eur Radiol. https://doi.org/10.1007/S00330-021-08021-6

3. Zawaideh JP, Sala E, Shaida N, et al (2020) Diagnostic accuracy of biparametric versus multiparametric prostate MRI: assessment of contrast benefit in clinical practice. Eur Radiol 30:. https://doi.org/10.1007/s00330-020-06782-0

4. van der Leest M, Israël B, Cornel EB, et al (2019) High Diagnostic Performance of Short Magnetic Resonance Imaging Protocols for Prostate Cancer Detection in Biopsy-naïve Men: The Next Step in Magnetic Resonance Imaging Accessibility. Eur Urol 76:574–581. https://doi.org/10.1016/j.eururo.2019.05.029

5. Alabousi M, Salameh JP, Gusenbauer K, et al (2019) Biparametric vs multiparametric prostate magnetic resonance imaging for the detection of prostate cancer in treatment-naïve patients: a diagnostic test accuracy systematic review and meta-analysis. BJU Int 124:209–220. https://doi.org/10.1111/BJU.14759

6. Greer MD, Shih JH, Barrett T, et al (2018) All over the map: An interobserver agreement study of tumor location based on the PI-RADSv2 sector map. J Magn Reson Imaging 48:482–490. https://doi.org/10.1002/JMRI.25948

7. Montagne S, Hamzaoui D, Allera A, et al (2021) Challenge of prostate MRI segmentation on T2-weighted images: inter-observer variability and impact of prostate morphology. Insights Imaging 12:. https://doi.org/10.1186/S13244-021-01010-9

8. Greer MD, Shih JH, Lay N, et al (2019) Interreader variability of prostate imaging reporting and data system version 2 in detecting and assessing prostate cancer lesions at prostate MRI. Am J Roentgenol 212:1197–1205. https://doi.org/10.2214/AJR.18.20536

9. Wang Y, Wang M (2020) Selecting proper combination of mpMRI sequences for prostate cancer classification using multi-input convolutional neuronal network. Phys Medica 80:92–100. https://doi.org/10.1016/J.EJMP.2020.10.013

10. Fernandez-Quilez A, Eftestøl T, Goodwin M, et al (2021) Self-transfer learning via patches: A prostate cancer triage approach based on bi-parametric MRI. Med Image Anal

11. Cuocolo R, Stanzione A, Castaldo A, et al (2021) Quality control and whole-gland, zonal and lesion annotations for the PROSTATEx challenge public dataset. Eur J Radiol 138:. https://doi.org/10.1016/J.EJRAD.2021.109647

12. de Rooij M, Israël B, Tummers M, et al (2020) ESUR/ESUI consensus statements on multi-parametric MRI for the detection of clinically significant prostate cancer: quality requirements for image acquisition, interpretation and radiologists’ training. Eur Radiol. https://doi.org/10.1007/s00330-020-06929-z

13. Barrett T, Padhani AR, Patel A, et al (2020) Certification in reporting multiparametric magnetic resonance imaging of the prostate: recommendations of a UK consensus meeting. BJU Int. https://doi.org/10.1111/bju.15285

14. Bass EJ, Pantovic A, Connor MJ, et al (2021) Diagnostic accuracy of magnetic resonance imaging targeted biopsy techniques compared to transrectal ultrasound guided biopsy of the prostate: a systematic review and meta-analysis. Prostate Cancer Prostatic Dis 2021 1–6. https://doi.org/10.1038/s41391-021-00449-7

15. Castiglioni I, Rundo L, Codari M, et al (2021) AI applications to medical images: From machine learning to deep learning. Phys Med 83:9–24. https://doi.org/10.1016/J.EJMP.2021.02.006

16. Schwier M, van Griethuysen J, Vangel MG, et al (2019) Repeatability of Multiparametric Prostate MRI Radiomics Features. Sci Rep 9:1–16. https://doi.org/10.1038/s41598-019-45766-z

17. Lee J, Steinmann A, Ding Y, et al (2021) Radiomics feature robustness as measured using an MRI phantom. Sci Reports 2021 111 11:1–14. https://doi.org/10.1038/s41598-021-83593-3

18. Dayan I, Roth HR, Zhong A, et al (2021) Federated learning for predicting clinical outcomes in patients with COVID-19. Nat Med 2021 2710 27:1735–1743. https://doi.org/10.1038/s41591-021-01506-3

19. Sathianathen NJ, Omer A, Harriss E, et al (2020) Negative Predictive Value of Multiparametric Magnetic Resonance Imaging in the Detection of Clinically Significant Prostate Cancer in the Prostate Imaging Reporting and Data System Era: A Systematic Review and Meta-analysis. Eur. Urol.

20. Vickers AJ, van Calster B, Steyerberg EW (2019) A simple, step-by-step guide to interpreting decision curve analysis. Diagnostic Progn Res 3:1–8. https://doi.org/10.1186/S41512-019-0064-7/FIGURES/3

21. Mongan J, Moy L, Kahn CE (2020) Checklist for Artificial Intelligence in Medical Imaging (CLAIM): A Guide for Authors and Reviewers. Radiol Artif Intell 2:e200029. https://doi.org/10.1148/RYAI.2020200029

22. Lambin P, Leijenaar RTH, Deist TM, et al (2017) Radiomics: the bridge between medical imaging and personalized medicine. Nat Rev Clin Oncol 2017 1412 14:749–762. https://doi.org/10.1038/nrclinonc.2017.141

23. QUADAS-2 | Bristol Medical School: Population Health Sciences | University of Bristol. https://www.bristol.ac.uk/population-health-sciences/projects/quadas/quadas-2/. Accessed 14 Oct 2021
